# Periostin promotes epithelial-mesenchymal transition via the MAPK/miR-381 axis in lung cancer

**DOI:** 10.18632/oncotarget.19273

**Published:** 2017-07-15

**Authors:** Wei-Wei Hu, Po-Chun Chen, Jun-Ming Chen, Yue-Ming Wu, Po-Yi Liu, Chih-Hao Lu, Yu-Feng Lin, Chih-Hsin Tang, Chia-Chia Chao

**Affiliations:** ^1^ Department of Thoracic Surgery, Dongyang People's Hospital, Dongyang, China; ^2^ Graduate Institute of Basic Medical Science, China Medical University, Taichung, Taiwan; ^3^ Department of Medical Research, Chung Shan Medical University Hospital, Taichung, Taiwan; ^4^ Department of Biotechnology, College of Health Science, Asia University, Taichung, Taiwan; ^5^ Graduate Institute of Biomedical Science, China Medical University, Taichung, Taiwan; ^6^ Department of Thoracic Surgery, Changhua Christian Hospital, Changhua, Taiwan; ^7^ Institute of Bioinformatics and Systems Biology, National Chiao Tung University, Hsinchu, Taiwan; ^8^ Department of Pharmacology, School of Medicine, China Medical University, Taichung, Taiwan; ^9^ Department of Respiratory Therapy, Fu-Jen Catholic University, New Taipei, Taiwan

**Keywords:** periostin, lung cancer, epithelial-mesenchymal transition, miR-381, MAPK

## Abstract

Periostin (POSTN, PN, or osteoblast-specific factor OSF-2) is a multifunctional cytokine that signals between the cell and the extracellular matrix. Periostin plays an important role in tumor development and is involved in carcinoma cell epithelial-mesenchymal transition (EMT), whereby mature epithelial cells undergo phenotypic morphological changes and become invasive, motile cells. Here, we discuss the molecular mechanisms involved in periostin-induced promotion of EMT in lung cancer cells. Online TCGA datasets demonstrate the prognostic relevance of periostin in lung cancer; a higher periostin level correlates with poor overall survival. Similarly, our IHC results show that high periostin expression is positively correlated with the EMT markers Snail and Twist, as well as stage of lung cancer. We found that recombinant periostin induces the EMT phenotype in lung cancer cells through the p38/ERK pathway, while pretreatment with chemical inhibitors prevented periostin-induced EMT induction. Moreover, we found that periostin regulates EMT by repressing microRNA-381 (miR-381) expression, which targets both Snail and Twist. Using the miR-381 mimic, we dramatically reversed periostin-induced Snail and Twist expression. Furthermore, periostin knockdown dramatically affected EMT markers and cell migration potential. The role of periostin in lung cancer progression is elucidated by the *in vivo* mouse model. Our findings indicate that changes in periostin expression in lung cancer may serve as a therapeutic target for the treatment of lung cancer metastasis.

## INTRODUCTION

Lung cancer is the most common cause of death from cancer worldwide. About 85% of all lung cancers are identified as non-small cell lung cancer (NSCLC) [1]. Currently, the 5-year survival rate is approximately 15% after diagnosis. Metastasis in late-stage lung cancer is responsible for the poor prognosis in this disease. As yet, the mechanisms of metastasis have not been fully elucidated.

EMT is now thought to be involved in disease states such as tissue fibrosis and cancer [2]. Much evidence illustrates how the EMT process provides a new basis for understanding invasive and metastatic behavior during cancer progression [3]. During EMT, epithelial cells lose their apical-basal polarity and transition to a mesenchymal phenotype [4]. The EMT of cancer cells is characterized by a downregulation of epithelial markers such as E-cadherin, α-cadherin, and γ-cadherin and acquisition of a mesenchymal phenotype, accompanied by upregulation of periostin, vimentin, fibronectin and N-cadherin. The overexpression of master regulators of EMT, such as Twist, Snail, Slug, ZEB1 and ZEB2, play a pivotal role in EMT regulation and promote early steps of metastasis [5]. These transcription factors suppress the expression of E-cadherin and in turn promote EMT [6]. Numerous studies have shown that lung cancer exhibits EMT-like states; EMT can occur in lung cancer cells upon activation of specific transcription factors such as Slug, which are associated with invasive behavior [7].

Periostin, also called osteoblast-specific factor 2 (OSF-2/POSTN), was first identified as a cell adhesion protein in a mouse osteoblastic cell line [8]. Recently, studies have demonstrated that periostin is a matricellular protein [9]. Periostin expression is required for bone, tooth and heart morphogenesis [10]. Periostin actively contributes to the pathobiology of inflammatory diseases, such as atherosclerosis, fibrosis and arthritis [11]. Moreover, periostin has been implicated in the metastatic process of many cancers, including for example breast cancer, colon cancer, head and neck cancer, ovarian cancer, and pancreatic ductal adenocarcinoma [9]. Recent investigations show that expression of periostin is highly correlated with metastasis, lymph node and lymphatic invasion in non-small cell lung cancer (NSCLC) [12]. Previous investigations have shown that transduction of periostin into non-metastatic 293T cells induces EMT by promoting cell migration, invasion, and adhesion [13]. Furthermore, periostin secreted from stromal cells promotes cell migration and correlates with the EMT process in human pancreatic cancer cells [14]. However, little is known about the mechanisms involved in periostin-induced EMT and metastatic progression in lung cancer. In the present study, we show that periostin promotes EMT in lung cancer and also Twist and Snail expression by inhibiting miR-381 via the ERK and p38 signaling pathways. This work provides a novel insight into the mechanism of periostin in lung cancer metastasis.

## RESULTS

### Periostin expression is positively correlated with Twist and Snail expression in lung cancer patient specimens

**Figure 1:**
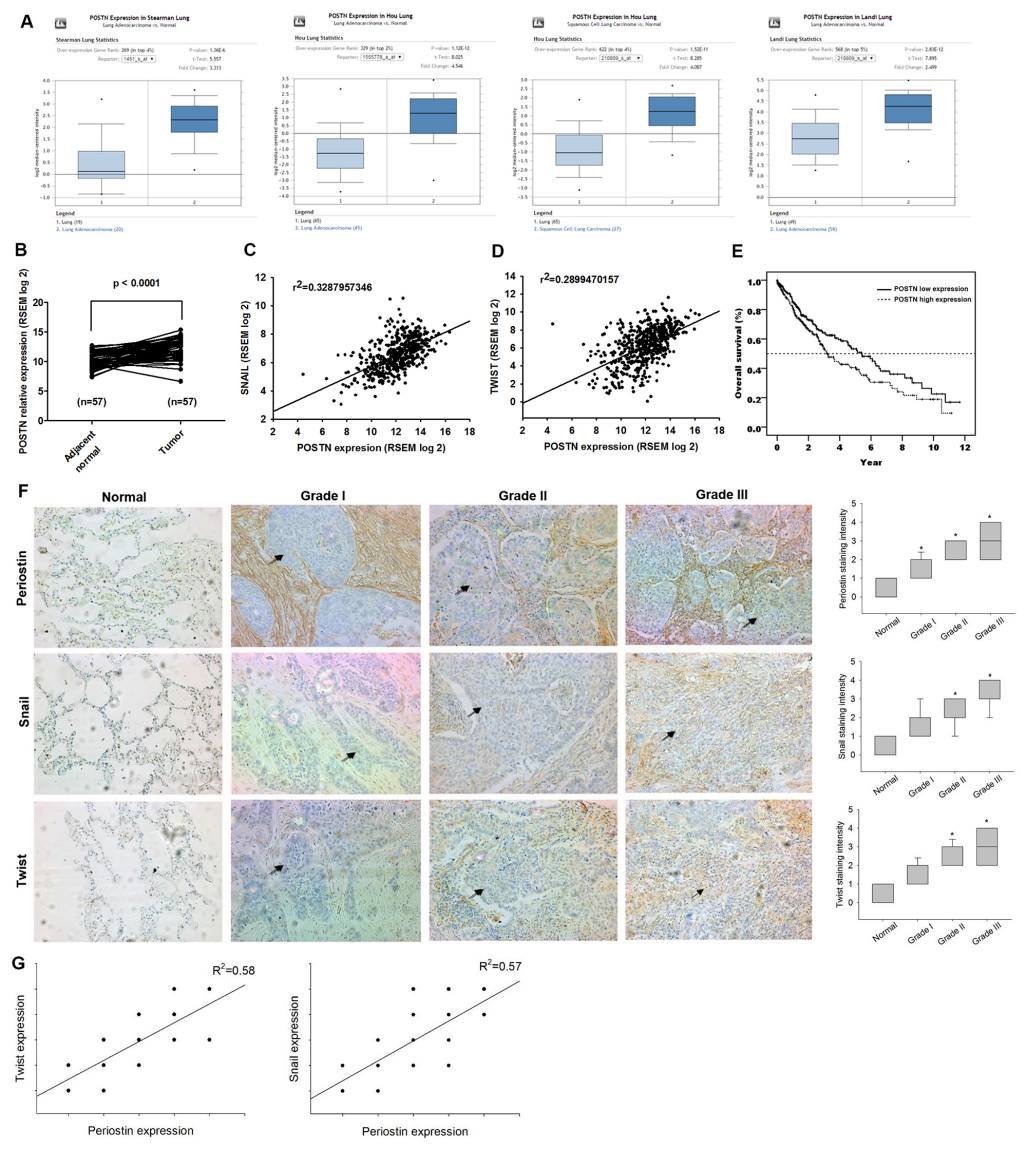
Periostin expression level and its prognostic value in lung cancer specimens **(A)** Periostin gene expression profiles in lung cancer specimens were obtained from the Oncomine database. Datasets in a single panel were from the same study. **(B)** Expression levels of periostin in paired adjacent normal and lung tumor tissues retrieved from TCGA dataset. **(C** and **D)** Correlation between periostin, Snail and Twist expression levels in lung cancer specimens retrieved from the TCGA dataset. **(E)** Kaplan-Meier analysis of overall survival, subgrouped by expression levels of periostin. **(F)** Lung cancer and normal tissue specimens were analyzed by IHC staining using periostin, Snail and Twist antibodies. The stained specimens were photographed by optical microscope and scored from 1-5 for expression levels. **(G)** Correlation between periostin, Snail and Twist expression levels in lung cancer specimens. *p < 0.05 compared with normal tissue specimens.

To investigate the role of periostin in lung cancer, we retrieved four datasets from the Oncomine database, which revealed that periostin expression levels were higher in tumor specimens than in normal tissues (Figure 1A). We also analyzed the TCGA dataset of lung squamous cell carcinoma, which revealed higher levels of periostin expression in tumor tissue compared with paired adjacent normal tissue (Figure 1B). Periostin is associated with EMT in embryogenesis and development [15]. However, it is unclear as to whether periostin regulates EMT in lung cancer, or serves as a novel marker in lung cancer prognosis. The basic helix-loop-helix transcription factor Twist and the zinc finger protein Snail have been identified as master regulators of EMT [16]. We therefore analyzed the correlation between expression levels of periostin, snail and twist from the TCGA dataset. The results indicated that periostin expression is positively correlated with Twist and Snail expression in lung cancer specimens (Fig. 1C and 1D). We then assessed the prognostic role of periostin in lung cancer by using the TCGA dataset. When the median was used as the cut-off value, higher periostin expression was associated with poor prognosis (p = 0.021) (Figure 1E). Our IHC results confirmed TCGA data, as we detected strong cytosolic staining of periostin in higher-grade tumor specimens. In addition, expression levels of periostin, Twist and Snail were positively correlated with tumor grade (Figure 1F) and periostin expression level was positively correlated with Twist and Snail expression in lung cancer specimens (Figure 1G).

### Periostin promotes EMT in lung cancer cells

**Figure 2:**
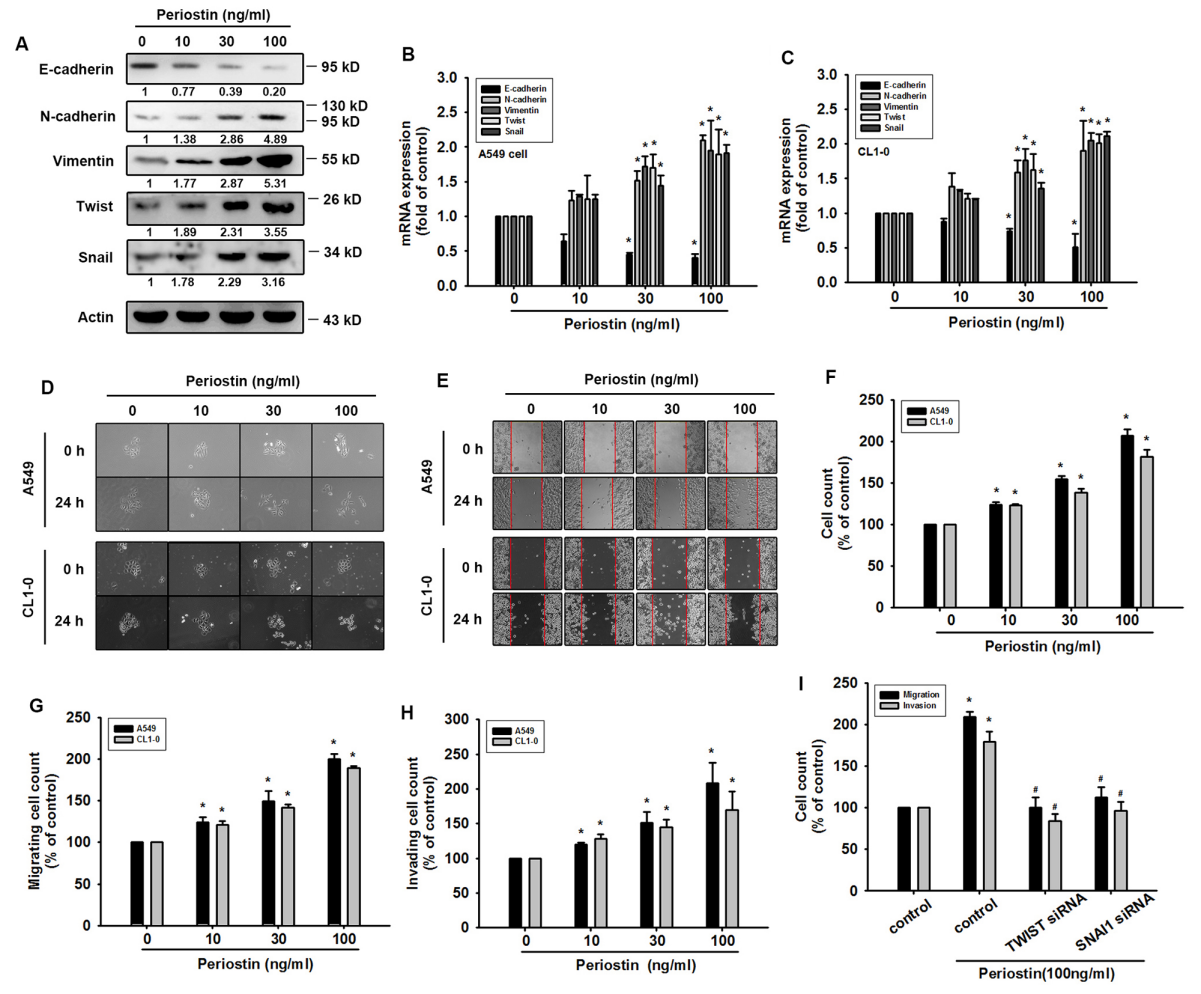
Periostin induces EMT in lung cancer cells **(A)** A549 cells were incubated with periostin (0-100 ng/ml) for 24 h. Western Blot analysis examined E-cadherin, N-cadherin, vimentin, Snail and Twist expression. Actin was used as an internal control. **(B** and **C)** A549 and CL1-0 cells were treated as described in (A), and E-cadherin, N-cadherin, vimentin, Snail and Twist expression was examined by qRT-PCR. **(D)** A549 and CL1-0 cells were incubated with periostin (0-100 ng/ml) for 24 h, and the scattering morphology was photographed. (E and F) A549 and CL1-0 cells were incubated with periostin (0-100 ng/ml) for 24 h, and *in vitro* migration was measured using the wound healing assay. **(G** and **H)** A549 and CL1-0 cells were treated with periostin (0-100 ng/ml) for 24 h, after which the Transwell assay was used to measure *in vitro* migration and the Matrigel invasion assay measured cell invasion. **(I)** A549 cells were transfected with Twist or Snail siRNA for 24 h, then incubated with periostin (100 ng/ml) for 24 h. *In vitro* migration was measured using the Transwell assay; cellular invasion was measured by the Matrigel invasion assay. Results are expressed as the mean ± S.E.M. *p < 0.05 compared with control. ^#^p < 0.05 compared with the periostin-treated group.

EMT has been linked to increased migration and invasiveness in the context of cancer [17]. To investigate the role of periostin in the process of EMT in lung cancer, we treated A549 and CL1-0 lung cancer cells with different doses of recombinant periostin. Following periostin treatment, qRT-PCR and Western blot analysis revealed induction of the EMT process by a shift in expression of cancer cells from epithelial (E-cadherin) to mesenchymal phenotypes (N-cadherin, vimentin, and Twist) (Figure 2A-2C). In previous studies, hepatocyte growth factor (HGF)-induced DU145 scatter models have been used to mimic conditions of the EMT phenomenon [18]. Similarly, our results showed that periostin treatment promoted scattering of the lung cancer cells in a dose-dependent manner after 24 h (Figure 2D). To confirm EMT phenotypes after periostin treatment, we assessed the mobility of lung cancer cells after periostin treatment in different assays (wound-healing, migration and invasion assays). The findings indicated that treatment with periostin promoted EMT in lung cancer cells (Figure 2E-2H, Supplementary Figure 1). Finally, lung cancer cells which were transfected with Twist and Snail siRNA inhibited periostin-promoted migration and invasion abilities (Figure 2I).

### ERK/p38 signaling pathways are involved in periostin-promoted EMT in lung cancer cells

**Figure 3:**
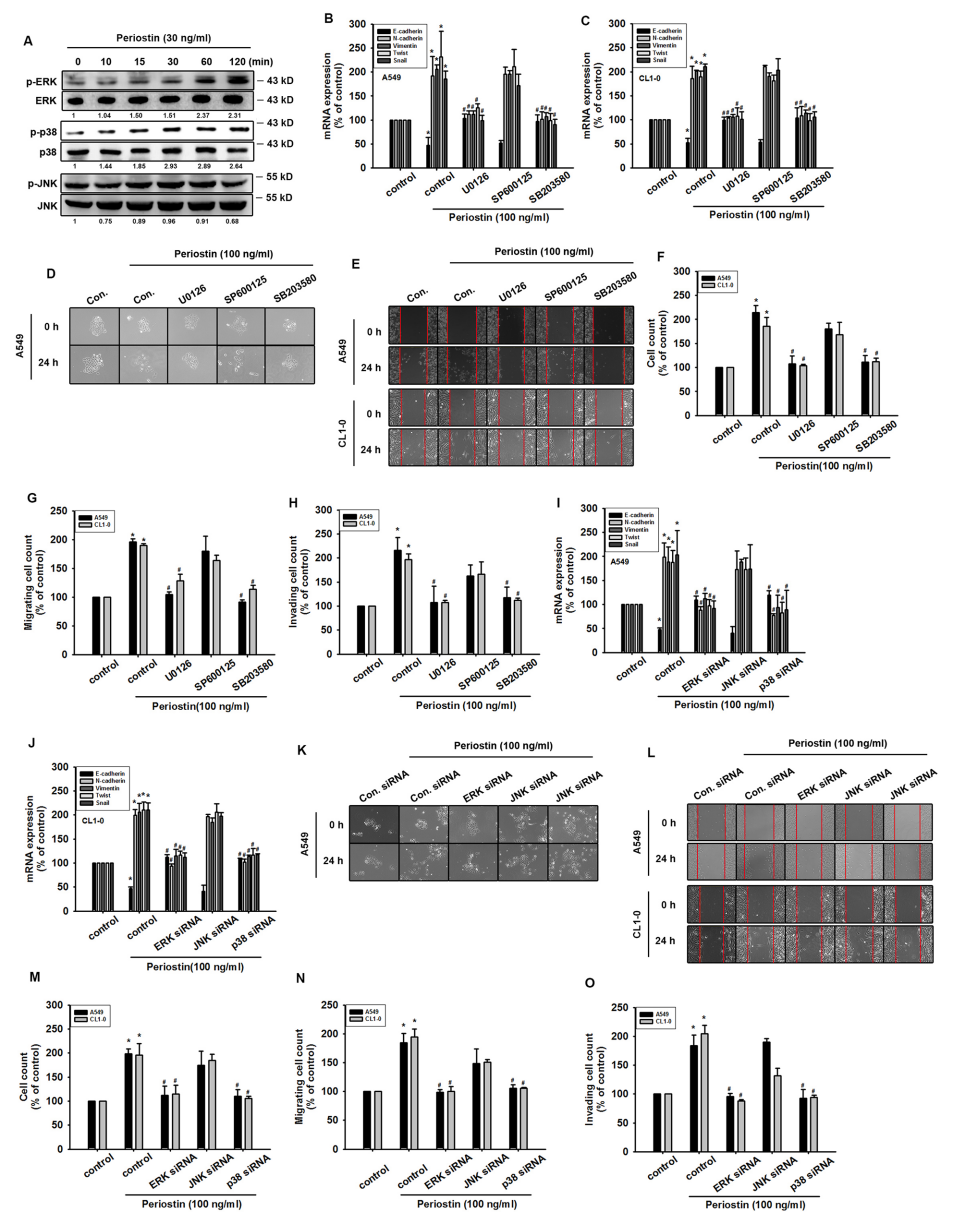
ERK and p38 signaling pathways are involved in periostin-promoted EMT in lung cancer cells **(A)** A549 cells were incubated with periostin (100 ng/ml) for the indicated times, and phosphorylation of EKR, p38 and JNK was determined by Western Blot analysis. **(B** and **C)** A549 and CL1-0 cells were pretreated with U0126 (10 μM), SB203580 (10 μM), or SP600125 (10 μM) for 30 min and then incubated with periostin (100 ng/ml) for 24 h, and expression levels of E-cadherin, N-cadherin, vimentin, Snail and Twist were examined by qRT-PCR. **(D)** A549 cells were treated as described in (B), and scattering morphology was photographed. **(E-H)** A549 and CL1-0 cells were treated as described in (B), and the wound healing assay, migration and invasion assays were assessed. **(I** and **J)** A549 and CL1-0 cells were transfected with ERK, p38 and JNK siRNA for 24 h, and incubated with periostin (100 ng/ml) for 24 h, while expression levels of E-cadherin, N-cadherin, vimentin, Snail and Twist were examined by qRT-PCR. **(K-O)** A549 and CL1-0 cells were treated as described in **(I)**, and scattering morphology, the wound healing assay, migration and invasion assays were assessed. Results are expressed as the mean ± S.E.M. *p < 0.05 compared with control. ^#^p < 0.05 compared with the periostin-treated group.

Previous work indicates that the MAPK pathway is a key mediator in EMT transcription factor activation [19]. Our results indicate that treatment of lung cancer cells with periostin (100 ng/ml) increases the phosphorylation of ERK and p38 signaling proteins but not that of JNK (Figure 3A). Moreover, whereas pretreatment with ERK and p38 inhibitors (U0126 and SB203580) reverse changes in periostin-promoted EMT markers in lung cancer cells, application of the JNK inhibitor (SP600125) has no such effect (Figure 3B and 3C).Pretreatment with ERK and p38 inhibitors inhibited scattering morphology, wound healing, migration and invasion potential of lung cancer cells (Figure 3D-3H, Supplementary Figure 2). When we transfected lung cancer cells with JNK, p38 and JNK siRNA, we found that periostin-induced promotion of the EMT process was blocked by ERK and p38 siRNAs, but not by the JNK siRNA (Figure 3I-3O).

### Periostin induces Twist and Snail expression by inhibiting miR-381 in lung cancer cells

**Figure 4:**
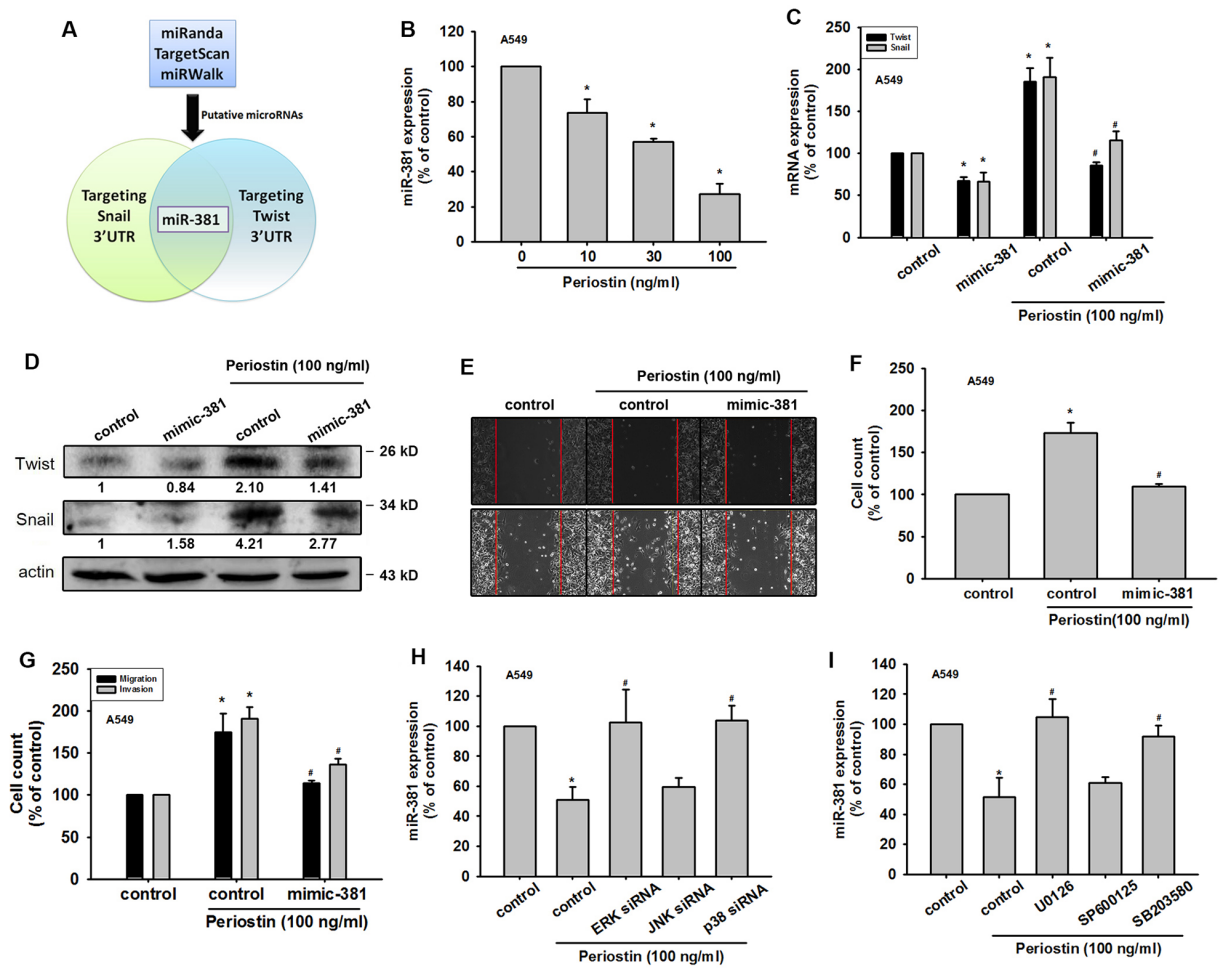
Periostin induces Twist and Snail expression by miR-381 repression in lung cancer cells **(A)** Schematic selection of candidate miRNAs. miR-381, which targets Twist and Snail 3’ UTR regions, was selected by using 3 online computational algorithms TargetScan, miRWalk and miRanda. **(B)** A549 cells were incubated with periostin (0-100 ng/ml) for 24 h. Total RNA was extracted and miR-381 expression was assessed by qRT-PCR. **(C-D)** A549 cells were transfected with control microRNA or miR-381 mimic for 24 h, then incubated with periostin for 24 h. Total RNA or protein was extracted, and Twist and Snail expression was assessed by qRT-PCR and Western Blot analysis. **(E-G)** A549 cells were transfected with miR-381 mimic or control mimic for 24 h, and incubated with periostin (100 ng/ml) for 24 h, and assessed by the wound healing assay, migration and invasion assays. **(H** and **I)** A549 cells were pretreated with U0126 (10 μM), SB203580 (10 μM), or SP600125 (10 μM) for 30 min or transfected with ERK, p38 and JNK siRNA for 24 h, then incubated with periostin (100 ng/ml) for 24 h. miR-381 expression was assessed by qRT-PCR. Results are expressed as the mean ± S.E.M. *p < 0.05 compared with control. #p < 0.05 compared with periostin-treated group.

Recent evidence has demonstrated the role played by miRNAs in modulating the metastatic process in the context of solid tumors [20]. Many studies have subsequently been conducted and a large number of miRNAs have been correlated with the EMT process [21]. Our results indicate that periostin induces the expression levels of Twist and Snail. We therefore used 3 online computational algorithms (TargetScan, miRanda and miRWalk) to explore candidate miRNAs that target Twist and Snail mRNA. Surprisingly, our results showed that miR-381 is the only microRNA to target the 3’-untranslated region (UTR) segments of both Twist and Snail mRNA (Figure 4A). We found that miR-381 expression was decreased in a dose-dependent manner after periostin treatment (Figure 4B). Moreover, periostin-induced Twist and Snail expression was abolished in A549 cells transfected with miR-381 mimic but not control miRNA (Figures 4C and 4D). We also confirmed the role of miR-381 in EMT by targeting Twist and Snail. The data indicated that the miR-381 mimic inhibited periostin-induced wound healing, migration and invasion potential (Figure 4E-4G). Finally, blocking of ERK and p38 signal cascades by inhibitors and siRNA dramatically reversed periostin-decreased miR-381 expression (Figure 4H and 4I).

### Knockdown of periostin expression represses *in vivo* tumor growth

**Figure 5:**
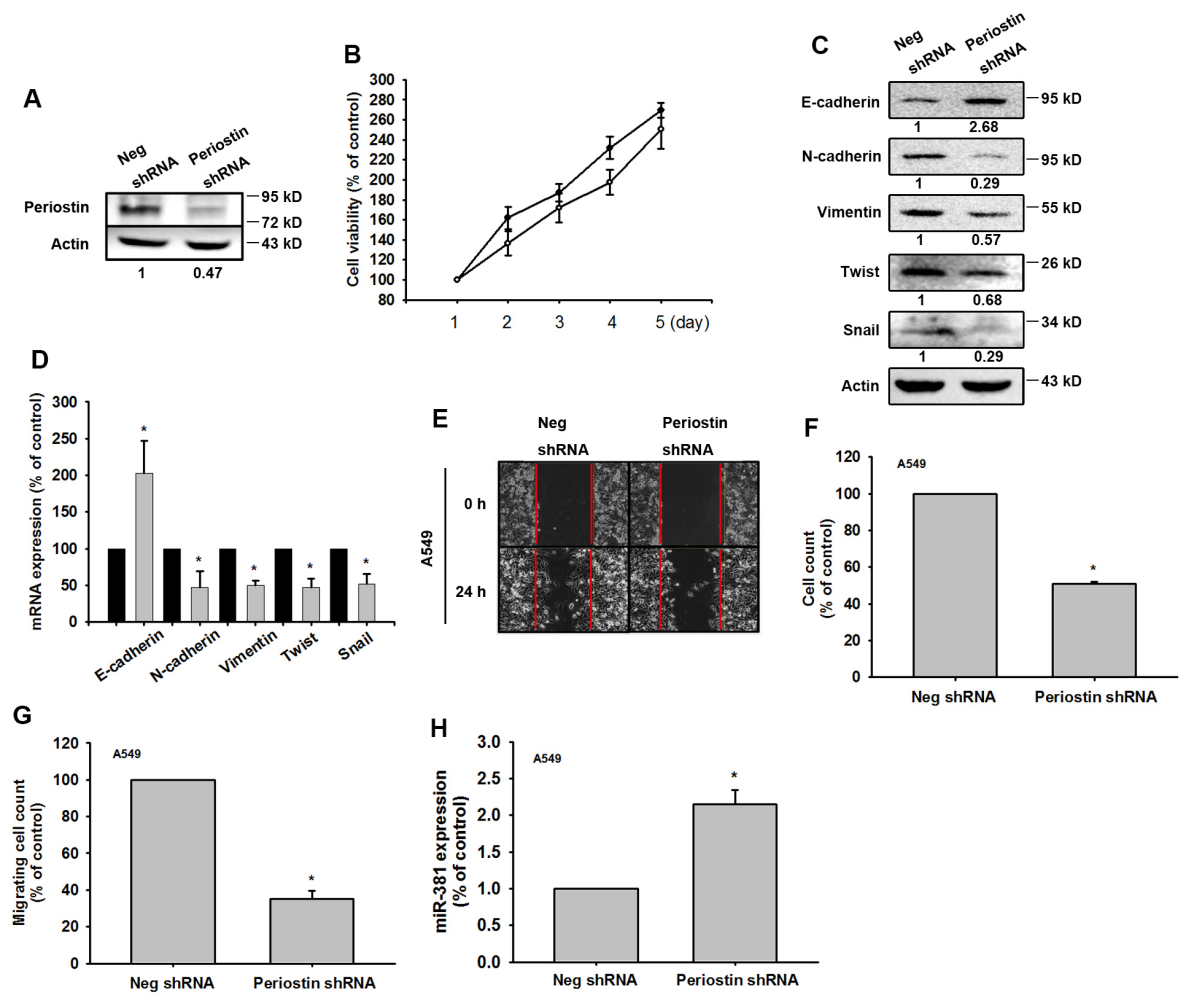
Knockdown of periostin expression represses EMT *in vitro* **(A)** Total protein was extracted from A549 cells stably expressing Neg shRNA and periostin shRNA. Expression levels of periostin were determined. **(B)** Cell proliferation was assessed by the MTT assay. **(C** and **D)** Protein and mRNA expression levels of E-cadherin, N-cadherin, vimentin, Twist and Snail were assessed by Western Blot and qRT-PCR. Actin was used as an internal control. **(E-G)** The wound healing and cell migration assays were performed in Neg shRNA and periostin shRNA cells. **(H)** miR-381 expression was assessed in Neg shRNA and periostin shRNA cells by qRT-PCR. Results are expressed as the mean ± S.E.M. *p < 0.05 compared with the Neg shRNA group.

To confirm the role of periostin in lung cancer metastasis, we examined A549 cells, which stably express periostin shRNA. We found that periostin levels were decreased in the periostin shRNA stable clone, without affecting cell proliferation (Figure 5A and 5B). We also found that EMT markers were affected by periostin knockdown (Figure 5C and 5D). In addition, periostin knockdown significantly reduced wound healing and migration ability in A549 cell lines (Figure 5E-5G). Levels of miR-381 expression were also affected by periostin knockdown (Figure 5H).

**Figure 6:**
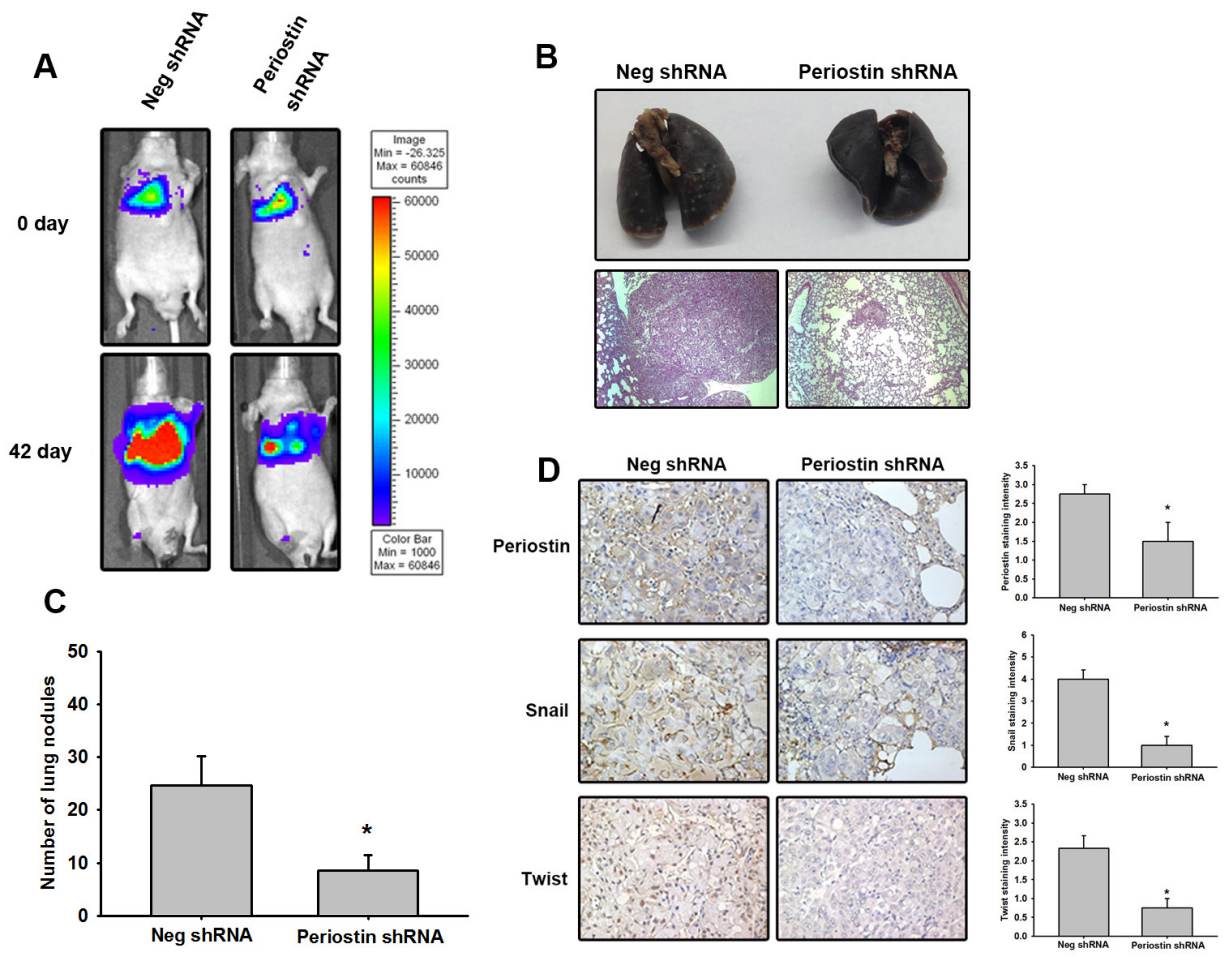
Knockdown of periostin expression represses tumor growth *in vivo* **(A)** Male nu/nu mice (6 weeks of age; N = 5 in each group) were intravenously injected with 5 × 10^5^ cells suspended in 50 μL PBS. Tumor growth was monitored using the IVIS protocol. The panels depict quantification of fluorescence imaging data obtained on days 1 and 42. **(B)** The mice were sacrificed on day 42. Lung sections were stained with H&E to monitor tumor growth. **(C)** The number of lung tumor nodules was counted under a dissecting microscope. **(D)** Lung specimens from sacrificed mice stained with periostin, Snail and Twist antibody. Stained specimens were photographed using an optical microscope (left panel). The staining intensity of periostin, Snail and Twist were scored from 1-5 to quantify the expression levels in lung specimens (right panel). Results are expressed as the mean ± S.E.M. *p < 0.05 compared with the Neg shRNA group.

To investigate the role of periostin in lung cancer progression, 4-week-old Nu/Nu mice (N = 5 in each group) were intravenously injected with A549 cells stably expressing luciferase. Tumors were monitored for size by the IVIS system at days 0 and 42. Periostin knockdown dramatically decreased tumor growth (Figure 6A). Mice were sacrificed at 42 days after tumor injection, and lungs were imaged using standard hematoxylin and eosin (H&E) and IHC staining protocols for histology. H&E findings indicated that tumor size was decreased in the periostin knockdown group (Figure 6B) as well as the number of nodules in tumor-bearing mice (Figure 6C), while IHC data showed low levels of periostin, Twist and Snail expression in the periostin knockdown group (Figure 6D).

## DISCUSSION

Periostin is a multifunctional cytokine that signals between the cell and the ECM. It is upregulated in a wide variety of cancers, including for example, NSCLC, breast cancer, colon cancer, head and neck cancer, ovarian cancer, and pancreatic ductal adenocarcinoma [9]. Here, we report for the first time the mechanisms by which periostin promotes EMT in lung cancer cells via the ERK/p38 pathway. We observed that ERK/p38 activation repressed miR-381 expression, which targets Twist and Snail. Our IHC data indicate that periostin expression is positively correlated with Twist, Snail and tumor stage in lung cancer. Our study provides a novel insight into the means by which periostin regulates the EMT process in lung cancer.

Periostin is an ECM protein that coordinates intercellular adhesion and elicits cell responses through interactions with ECM proteins, for example fibronectin, tenascin-C and collagen V [22, 23]. Previous research has found that periostin is highly expressed in various tumors, such as oral cancer [24], NSCLC [25], prostate cancer [26] and breast cancer [27]. Overexpression of periostin in non-metastatic 293T cells enables them to develop the EMT phenotype and thus promote invasion and metastasis *in vivo* [13]. The correlation between periostin expression and EMT has been observed in prostate cancer, through the downregulating of E-cadherin expression via Snail [28]. Here, we investigated the mechanisms involved in periostin-induced EMT in NSCLC. We have demonstrated that recombinant periostin promoted the EMT process in lung cancer cells in a dose-dependent manner (10-100 ng/ml). Healthy controls have serum periostin concentrations of around 70 ng/ml [29]. In our study, higher concentrations of periostin effectively promoted tumor progression. In another study, periostin concentrations of 50 or 100 ng/ml increased migration and proliferation of human periodontal ligament fibroblasts [30], which is in agreement with our findings. Previous investigations into periostin expression levels in NSCLC clearly show that higher periostin expression correlates positively with poor prognosis [31]. Similarly, Takanami et al. describe a positive correlation between higher periostin expression with microvessel density and lymphatic microvessel density [12]. All of these study findings highlight the critical role played by periostin in angiogenesis and lymphangiogenesis [31]. Furthermore, these studies show that this involvement of periostin in tumor progression is a multimechanistic process. Previous investigations using the monoclonal periostin-blocking antibody OC-20 in a murine model of human melanoma show that OC-20 significantly inhibits tumor growth and angiogenesis [32]. Periostin monoclonal antibody treatment may therefore have potential in lung cancer.

Recent research indicates that periostin activates ERK signaling and promotes the cancer stem cell phenotype [33]. This is not unexpected, considering that analogies exist between the EMT process and cancer stem cell phenotype. The emergence of the cancer stem cell phenotype occurs in part as a result of the EMT process [34]. MAPK signaling has been implicated in EMT regulation. Many growth factors such as HGF, EGF, and TGF-β which elicit EMT are able to activate the MAPK signaling pathway [3]. Our present work describes the central role of the MAPK signaling pathway in regulating periostin-induced EMT. Further research is needed to clarify the receptors that mediate this activation.

Recently, several reviews have indicated that microRNAs serve as regulators in the EMT process. Our data show that the miR-381 mimic represses both Snail and Twist expression (Figure 4C and 4D). Moreover, miR-381 abolishes EMT-related events such as cell migration, invasion and wound healing potential (Figure 4E-4G). These results indicate that miR-381 is an oncogenic microRNA. As shown in other studies, miR-381 expression levels are downregulated in various tumors, such as in epithelial ovarian cancer tissue. miR-381 inhibits epithelial ovarian cancer cell proliferation, migration and invasion, via suppression of its target gene, YY1 [35]. Recent research demonstrates that miR-381 overexpression inhibits hepatocellular carcinoma cell growth and invasion by targeting the liver receptor homolog-1 [36]. Here, we are the first to report that miR-381 regulates EMT by repressing Snail and Twist expression. As previous studies have suggested, miRNA-381 may be a promising new target in lung cancer treatment.

Although we have described the signaling cascade involved in the periostin-induced promotion of EMT, a limitation of our study is that we do not provide evidence on the receptor that mediates periostin activation. Previous investigations indicate that the promotion of EMT by periostin requires cross-talk between integrin and EGFR signaling pathways [13]; this remains to be clarified. In conclusion, our study elucidates the mechanism of periostin-induced EMT in lung cancer; miR-381 may play a pivotal role in this process. Our finding provides a novel insight into the role of periostin in cancer metastasis and indicates that periostin may potentially be a useful target for metastasis treatment.

## MATERIALS AND METHODS

### Material

Protein A/G beads, anti-mouse and anti-rabbit IgG-conjugated horseradish peroxidase, rabbit polyclonal antibodies specific for p38 (SC-535; Santa Cruz, TX, USA), p-p38 (SC-166182; Santa Cruz), ERK (SC-154; Santa Cruz), p-ERK (SC-7383; Santa Cruz), JNK (SC-7345; Santa Cruz), p-JNK (SC-6254; Santa Cruz), E-cadherin (ab40772; Abcam, Cambridge, UK), N-cadherin (ab76057; Abcam), vimentin (SC-6260; Santa Cruz), Twist (SC-81417; Santa Cruz), β-actin (sc-130656; Santa Cruz) or periostin (SC-67233; Santa Cruz) were purchased from Santa Cruz Biotechnology (Santa Cruz, TX, USA) or Abcam (Cambridge, UK). ON-TARGETplus siRNAs of Twist (L00643400; Dharmacon), Snail (L01084701; Dharmacon), ERK (L00355500; Dharmacon), p38 (L00351200; Dharmacon), JNK (L00351400; Dharmacon) and control were purchased from Dharmacon Research (Lafayette, CO, USA). Recombinant human periostin was purchased from PeproTech (Rocky Hill, NJ, USA). miRNA control and miR-381 mimic were purchased from Invitrogen (Carlsbad, CA, USA). The short hairpin RNA (shRNA) plasmid used for gene knockdown studies was purchased from National RNAi Core Facility Platform (Taipei, Taiwan). All other chemicals were obtained from Sigma–Aldrich (St Louis, MO , USA).

### Data retrieval from the online oncomine database

The periostin mRNA expression patterns of NSCLC datasets were retrieved from the Oncomine cancer gene expression microarray database (DB) (https://www.oncomine.org/resource/login.html). The Stearman Lung [37], Hou Lung [38] and Landi Lung [39] were retrieved for further analysis.

### Analysis of the cancer genome atlas (TCGA) dataset

A total of 504 lung squamous cell carcinomas were retrieved from the TCGA, which contained mRNA sequencing and clinical data. Fifty-seven paired normal and tumor specimens were used to analyze periostin expression. Paired t tests were performed and a p-value < 0.05 showed significance. mRNA sequencing results from the dataset were used to analyze correlations between periostin, Snail and Twist. The overall survival analysis of the TCGA dataset were performed using the Kaplan-Meier analysis module of the IBM SPSS Statistics software (IBM Corp., Armonk, NY, USA). The difference between the two groups was compared by the log rank test.

### Cell culture

The human lung adenocarcinoma cell line A549 was obtained from the American Type Culture Collection (ATCC; Manassas, VA, USA). Human lung adenocarcinoma cells CL1-0 and its subclone CL1-5, which has higher invasiveness, were kindly gifted by Dr. Shun-Fa Yang (Institute of Medicine, Chung Shan Medical University, Taichung, Taiwan). A549 cells were cultured in F12-K medium and DMEM: F12 (1:1 mix) respectively; CL1-0 and CL1-5 cell lines were cultured in α-MEM medium. All cells were supplemented with 20 mM HEPES, 10% FBS, 2 mM glutamine, penicillin, and streptomycin (Invitrogen, Carlsbad, CA), and maintained at 37°C in a 5% CO2 atmosphere.

### Scatter assay

To monitor EMT changes *in vitro*, the cell scatter assay was performed on A549 cells. Cells were seeded in a 24-well plate at a density of 0.5 × 10^4^ cells/well. The cells formed small colonies after 48 h. Subsequently, the cells were treated and incubated for a further 24 h, as described in the legends in Figures 2 and 3. Finally, cells were fixed in 4% PFA and photographed by microscope-mounted camera.

### Wound healing assay

For wound-healing migration assays, cells were seeded on 12-well plates at a density of 1 × 10^5^ cells/well in culture medium. At 24 h after seeding, the confluent monolayer of culture was scratched with a fine pipette tip, and migration was visualized by microscopy. The rate of wound closure was observed at the indicated times.

### Western blot analysis

The cellular lysates were prepared and proteins were then resolved on SDS–PAGE and transferred to Immobilon polyvinyldifluoride (PVDF) membranes. The blots were blocked with 4% BSA for 1 h at room temperature and then probed with rabbit or mouse anti-human antibodies against periostin, vimentin, N-cadherin, E-cadherin, Twist, Snail, p-ERK, ERK, p-p38, p38, p-JNK, JNK, and β-actin (1:1000) for 1 h at room temperature. After three washes, the blots were subsequently incubated with a donkey anti-rabbit peroxidase-conjugated secondary antibody (1:1000) for 1 h at room temperature. The protein bands were visualized by enhanced chemiluminescence using ImageQuant LAS 4000 (GE Healthcare Life Sciences, Little Chalfont, UK). Quantitative data were obtained using a computing densitometer and ImageQuant software (Molecular Dynamics, Sunnyvale, CA, USA).

### Quantitative real time PCR

Quantitative real time PCR (qRT-PCR) analysis was carried out using the Taqman® one-step PCR Master Mix (Applied Biosystems, Foster City CA). 100 ng of total cDNA was added per 25 μl reaction with sequence-specific primers and Taqman® probes. Sequences for all target gene primers and probes were purchased commercially (β-actin was used as the internal control) (Applied Biosystems, CA). qRT-PCR assays were carried out in triplicate on a StepOnePlus sequence detection system. The cycling conditions were 10 min polymerase activation at 95°C followed by 40 cycles at 95°C for 15 sec and 60°C for 60 sec. The threshold was set above the non-template control background and within the linear phase of target gene amplification to calculate the cycle number at which the transcript was detected (denoted as C_T_).

### miRNA qRT-PCR analysis

Total RNAs were extracted and cDNA was synthesized using the Mir-X™ miRNA First-Strand Synthesis Kit (Clontech, CA, USA). qRT-PCR assays were carried out in triplicate on a StepOnePlus sequence detection system. The cycling conditions were 10 min polymerase activation at 95°C followed by 40 cycles at 95°C for 15 sec and 60°C for 60 sec. Relative gene expression was quantified using an endogenous control gene (U6). The threshold cycle (CT) was defined as the fractional cycle number at which fluorescence passed a fixed threshold, and relative expression was calculated using the comparative CT method.

### Transwell migration and invasion assay

All cell migration assays were performed using Transwell inserts (8-μm pore size; Costar, NY) in 24-well dishes. The lung cancer cells were pretreated for 30 min with the indicated concentrations of inhibitors or vehicle (0.1% DMSO). Cells (1 × 10^4^ in 200 μl of serum-free medium) were then seeded in the upper chamber of the Transwell and 300 μl of the same medium containing varying concentrations of periostin (R&D Systems, Minneapolis, MN, USA) was placed in the lower chamber. Each experiment was performed with triplicate wells and repeated at least 3 times. For the cell invasion assay, each well was pre-coated with Matrigel (25 mg/50 mL; BD Biosciences, Bedford, MA) to form a continuous, thin layer. Protocol followed the migration assay as described below.

### Periostin knockdown stable clone in lung cancer cells

For periostin knockdown, the periostin shRNA plasmid was purchased from the National RNAi Core Facility (RNAi Core, Academia Sinica, Taiwan). Empty vector was used as a negative control. The high malignancy lung cancer cell line A549 was transduced with luciferase, to enable monitoring of tumor growth using *In Vivo* Imaging Systems (IVIS, Xenogen, UK). Following luciferase transfection, stable cells were transfected with periostin shRNA plasmid. Puromycin was used for screening cells; surviving cells were used as stable periostin knockdown cell lines.

Cell viability was assessed by the 3-(4,5-dimethylthiazol-2-yl)-2,5- diphenyltetrazolium bromide (MTT) assay. The negative vector and periostin knockdown stable cells were seeded in 6 well plates and grown for 1 to 5 days. The cells were washed with PBS and incubated with MTT (0.5 mg/mL) at 37 °C for 2 h. After 2 h, cells were incubated with DMSO to dissolve formazan crystals. After the mixture was shaken at room temperature (RT) for 10 min, absorbance of each well was determined at 550 nm using a microplate reader (Bio-Tek, Winooski, VT, USA).

### Animal model and imaging procedures

All experimental procedures were approved by the Institutional Animal Care and Use Committee of China Medical University. All mice were maintained under a specific pathogen-free environment in the animal research center of China Medical University and all experiments were performed in accordance with relevant guidelines and regulations for animal care and use. Male nu/nu mice (6–8 weeks of age) were administered intravenous injections of 5× 10^5^ cells suspended in 50 μL PBS. After injection, tumor growth and local metastasis was monitored using the *In Vivo* Imaging System (IVIS, Xenogen, UK). The mice were sacrificed after 42 days. Lungs of the sacrificed mice were dissected and subjected to IHC staining.

### Histology and immunohistochemistry

Human lung cancer tissue arrays (LC242a and LC1921) were purchased from Biomax (Rockville, MD). All specimens in these arrays contain 170 cases of NSCLC tissue (90 cases of squamous cell carcinoma and 80 cases of adenocarcinoma), plus 14 adjacent normal lung tissue and 20 normal lung tissue cases. The NSCLC tissues contained 25 Grade I, 86 Grade II and 44 Grade III tissue specimens, as defined by the histological grading system provided by Biomax.

Lung tissue samples were collected from sacrificed mice, fixed in 4% paraformaldehyde in PBS for at least 72 h, and dehydrated in increasing concentrations of ethanol. All samples were then embedded in paraffin. Serial sections of 5-μm thicknesses were cut longitudinally for staining with standard hematoxylin-eosin (H & E). For IHC staining, the tissue was placed on glass slides, rehydrated, incubated with 3% hydrogen peroxide to quench endogenous peroxidase activity, and then blocked by 3% BSA incubation in PBS. The tissues were incubated with primary mouse anti-human periostin, Snail and Twist antibody at 1:100 dilutions, at 4°C overnight. After undergoing three PBS washes, tissues were incubated with a biotin-labeled secondary antibody. The Vector Laboratories ABC Kit (Vector Laboratories, Burlingame, CA) was used to perform immunohistochemistry in tissue sections. Slides were stained with the chromogen diaminobenzidine, washed, counterstained with Delafield’s hematoxylin, dehydrated, treated with xylene, then mounted. Stained specimens were photographed by microscope. IHC stains for periostin, Snail and Twist were scored from 0-5 to quantify the expression levels observed in the photographs, using the following scoring approach: 0 = no staining or unspecific staining of tumor cells; 1 = very weak (intensity) of tumor cells; 2 = weak staining of tumor cells; 3 = moderate staining of tumor cells; 4 = strong staining of tumor cells; 5 = very strong staining of tumor cells. A pathologist evaluated the spectrum of staining intensity in all the samples and then arbitrarily categorized the staining intensity into the various scores based on the overall staining.

### Statistics

The values given are means ± S.E.M. The significance of difference between the experimental groups and controls was assessed by the Student’s *t* test. The overall survival was performed by Kaplan-Meier method. The difference in survival between the groups was compared by the log rank test. The difference was significant if the *p* value was <0.05.

## SUPPLEMENTARY MATERIALS FIGURES



## Abbreviations

EMT: epithelial-mesenchymal transition
Periostin: POSTN
microRNA-381: miR-381
NSCLC: non-small cell lung cancer
IHC: immunohistochemistry
PVDF: polyvinyldifluoride
TCGA: The Cancer Genome Atlas
