# Supplementary material for: Periostin promotes epithelial-mesenchymal transition via the MAPK/miR-381 axis in lung cancer
Source: Oncotarget. 2017 Jul 15;8(37):62248–60. doi: 10.18632/oncotarget.19273 (PMC5617502; doi:10.18632/oncotarget.19273)
Supplement: Supplementary file 1 [file oncotarget-08-62248-s001.pdf]

Periostin promotes epithelial-mesenchymal transition via the MAPK/miR-381 axis in lung cancer

SUPPLEMENTARY MATERIALS

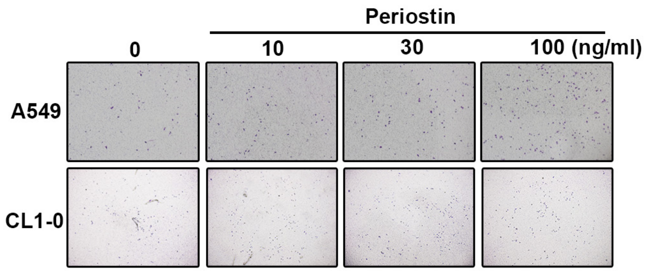

Supplementary Figure 1: Periostin induces EMT in lung cancer cells. A549 and CL1-0 cells were treated with periostin (0-100 ng/ml) for 24 h, after which the Transwell assay was used to measure in vitro migration.

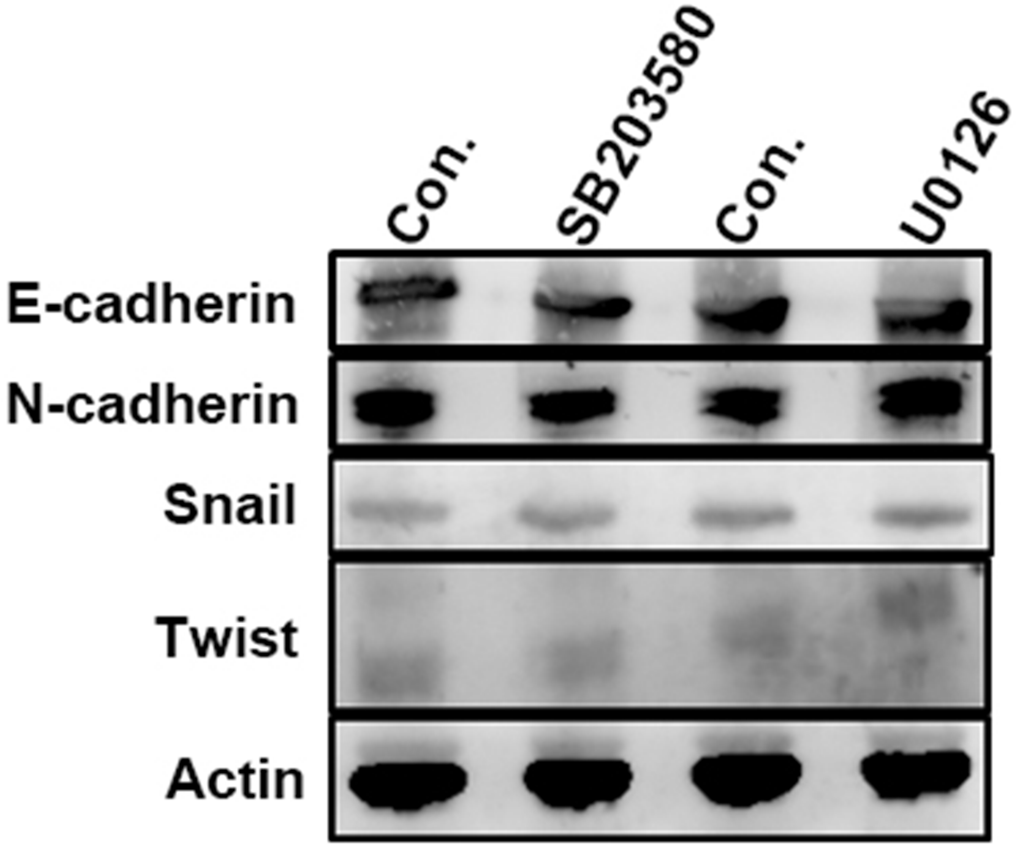

Supplementary Figure 2: The efficacy of p38 and ERK inhibitors on EMT markers expression. The A549 cells were treated with U0126 (10 µM) and SB203580 (10 µM) for 24 h, and expression levels of E-cadherin, N-cadherin, Snail and Twist were examined by Western Blot.
